# Use of mHealth Technology for Improving Exercise Adherence in Patients With Heart Failure: Systematic Review

**DOI:** 10.2196/54524

**Published:** 2025-01-09

**Authors:** Pallav Deka, Erin Salahshurian, Teresa Ng, Susan W Buchholz, Leonie Klompstra, Windy Alonso

**Affiliations:** 1 College of Nursing Michigan State University East Lansing, MI United States; 2 College of Nursing University of Nebraska Medical Center Omaha, NE United States; 3 Department of Health, Medicine and Care Sciences Linkoping University Linkoping Sweden

**Keywords:** adherence, activity monitors, exercise, heart failure, mHealth, mobile health, smartphone, videoconferencing, heart, mHealth technology, exercise programs, age, sex, race, telehealth technology, software apps, feasibility, mobile phone

## Abstract

**Background:**

The known and established benefits of exercise in patients with heart failure (HF) are often hampered by low exercise adherence. Mobile health (mHealth) technology provides opportunities to overcome barriers to exercise adherence in this population.

**Objective:**

This systematic review builds on prior research to (1) describe study characteristics of mHealth interventions for exercise adherence in HF including details of sample demographics, sample sizes, exercise programs, and theoretical frameworks; (2) summarize types of mHealth technology used to improve exercise adherence in patients with HF; (3) highlight how the term “adherence” was defined and how it was measured across mHealth studies and adherence achieved; and (4) highlight the effect of age, sex, race, New York Heart Association (NYHA) functional classification, and HF etiology (systolic vs diastolic) on exercise adherence.

**Methods:**

We searched for papers in PubMed, MEDLINE, and CINAHL databases and included studies published between January 1, 2015, and June 30, 2022. The risk of bias was analyzed.

**Results:**

In total, 8 studies (4 randomized controlled trials and 4 quasi-experimental trials) met our inclusion and exclusion criteria. A moderate to high risk of bias was noted in the studies. All studies included patients with HF in NYHA classification I-III, with sample sizes ranging from 12 to 81 and study durations lasting 4 to 26 weeks. Six studies had an equal distribution of male and female participants whose ages ranged between 53 and 73 years. Videoconferencing was used in 4 studies, while 4 studies used smartphone apps. Three studies using videoconferencing included an intervention that engaged participants in a group setting. A total of 1 study used a yoga program, 1 study used a walking program, 1 study combined jogging with walking, 1 study used a cycle ergometer, 2 studies combined walking with cycle ergometry, and 1 study used a stepper. Two studies incorporated resistance exercises in their program. Exercise programs varied, ranging between 3 and 5 days of exercise per week, with exercise sessions ranging from 30 to 60 minutes. The Borg rating of perceived exertion scale was mostly used to regulate exercise intensity, with 3 studies using heart rate monitoring using a Fitbit. Only 1 study implicitly mentions developing their intervention using a theoretical framework. Adherence was reported to the investigator-developed exercise programs. All studies were mostly feasibility or pilot studies, and the effect of age, sex, race, and NYHA classification on exercise adherence with the use of mHealth was not reported.

**Conclusions:**

The results show some preliminary evidence of the feasibility of using mHealth technology for building exercise adherence in patients with HF; however, theoretically sound and fully powered studies, including studies on minoritized communities, are lacking. In addition, the sustainability of adherence beyond the intervention period is unknown.

## Introduction

With the aging population, heart failure (HF) is a growing problem worldwide [1]. In the United States, 8 million individuals are projected to be diagnosed with HF, and the cost of its treatment is expected to reach US $70 billion by 2030 [2-4]. Irrespective of the etiology of the disease (HF with preserved vs reduced ejection fraction; ischemic vs nonischemic), patients with HF experience symptoms of fatigue, exercise intolerance, palpitations, and over time, experience a decline in physical functioning [5-10]. These symptoms worsen as the disease progresses, often designated by the New York Heart Association (NYHA) I-IV classification [11]. The treatment and management of HF are also complicated by patients experiencing peaks and troughs in symptom experience and often needing hospitalizations. HF is generally associated with advancing age and has the highest readmission rates among all chronic diseases, adding to the increase in health care costs [2,12]. As such, effective and efficient management of HF using both pharmacological and nonpharmacological methods is essential.

As a nonpharmacological method, exercise training interventions have been shown to decrease hospitalizations, increase exercise capacity, and improve quality of life [13]. Exercise is different from physical activity and has been defined as a subset of physical activity that is planned, structured, purposeful, and performed with the objective of improving physical fitness [14]. The landmark multisite Heart Failure: A Controlled Trial Investigating Outcomes of Exercise Training trial established the safety of moderate-intensity aerobic exercise in HF and found evidence of reduced rehospitalization rates in patients who were adherent versus nonadherent to the recommended exercise [15]. Based on clinical evidence, exercise is considered a class I recommendation in adults with HF, meaning that its benefits greatly outweigh the risks [16-18]. Current guidelines recommend that patients with HF continue to include 150 minutes per week of moderate-intensity aerobic exercise supplemented with 2-3 days of resistance exercise training [19]. However, desired adherence to the recommended exercise in this population has been hard to achieve [15,20,21]. The World Health Organization [22] defines adherence as “the extent to which a person’s behavior—taking medication, following a diet, and/or executing lifestyle changes, corresponds with agreed recommendations from a health care provider.” This definition differentiated adherence from compliance and attempted to highlight the importance and need for lifestyle and behavioral changes to successfully achieve adherence [23].

In recent years, advances in mobile health (mHealth) technology have provided the opportunity for bridging access to care by delivering interventions to participants’ homes using video calling, chatbot messaging, cloud-based digital voice response, automated emails, phone counseling, and other tools for motivation and engagement [24]. Such technologies have also significantly improved the ability to remotely monitor a person's health status and intervention outcomes [24]. A systematic review of reviews shows that their app has also been used in the management of various health conditions such as asthma, chronic lung disease, diabetes, cardiac rehabilitation, hypertension, and HIV management, among many others [24]. Additionally, mHealth has also been implemented for behavioral or lifestyle changes that include weight loss, physical activity, smoking cessation, and sexual behavior [24]. Across diverse clinical populations, the effectiveness of home-based exercise programs is found to be similar to that of facility-based exercise programs [25]. In our previous study published in 2017 [26], we updated the state of the science from 2009 [27] in relation to adherence to exercise in patients with HF. In that systematic review, we found a mix of home-based and facility-based interventions and highlighted the lack of web-based interventions to improve adherence to recommended exercise guidelines in this population [26]. We did not find any studies at that time with mHealth interventions specifically targeting and reporting exercise adherence in HF. With significant advances in mHealth technology and access to smartphones among all ages [28], it is important to update the literature and summarize the work that has been done since to improve adherence to exercise in patients with HF using newer technologies. Demographic and socioeconomic variables can have a profound impact on technology adoption and use [29]. There can be unique challenges for implementing an mHealth intervention depending on the type of technology used and the complexity of the exercise program. This review was performed to systematically synthesize the literature on the use of mHealth technology that reported adherence to exercise in patients with HF, the majority of whom tend to be older adults. Specifically, this review was done to (1) describe study characteristics of mHealth interventions for exercise adherence in HF including details of sample demographics, sample sizes, exercise program, and theoretical frameworks; (2) summarize types of mHealth technology used to improve exercise adherence in patients with HF; (3) highlight how the term “adherence” was defined and how it was measured across mHealth studies and adherence achieved; and (4) highlight the effect of age, sex, race, NYHA functional classification, and HF etiology (systolic vs diastolic) on exercise adherence. Finally, we conclude by summarizing our findings and providing suggestions for future studies.

## Methods

### Study Design

The PRISMA (Preferred Reporting Items for Systematic Review and Meta-Analyses) statement guided this review [30]. A health science librarian worked closely with the lead authors (PD, WA, ES, and TN) on narrowing the search terms. The search strategy included the terms (“heart failure” AND “exercise” AND “adherence”), AND (“mhealth” OR “telehealth”) in the title or abstract. Three databases (PubMed, MEDLINE, and CINAHL) were searched. Studies were eligible if they met the criteria outlined in Textbox 1. These inclusion dates were chosen because a previous systematic review in the same topic area had included studies published up to 2015 [26].

**Inclusion and exclusion study criteria.**

**Inclusion criteria**

- Primary studies on patients with heart failure
- Used an intervention that included mobile health or telehealth technology
- Experimental or quasi-experimental study
- Primary or secondary outcome reports of exercise adherence
- Published between January 1, 2015, and June 30, 2022
- Published in the English language

**Exclusion criteria**

- Studies that used only cell phone text messaging as an intervention

### Screening and Data Extraction

All papers obtained from the literature search were uploaded and screened using the Rayyan program. The following 2-step screening process was used: (1) 4 authors (PD, WA, ES, and TN) independently screened titles and abstracts, and (2) all 4 authors screened the full texts of the included papers from step 1 to be included in this review. During every step, the 4 authors labeled a paper as “accepted,” indicating that the paper met inclusion criteria; “not accepted,” indicating that the paper did not meet inclusion criteria; or “maybe,” indicating that the paper needed to be discussed prior to making a judgment that the paper was acceptable for the next step. As a group, the 4 authors discussed discrepancies and “maybe” papers to include or not include in the final analysis.

An author-developed extraction method was developed according to our prior systematic review [26]. Data including study characteristics (country, study design, theoretical framework, and length of study), sample characteristics (sample size and HF sample characteristics), intervention characteristics (mHealth technology and intervention components), outcomes (primary outcome, secondary outcomes, and outcome measures), results, and conclusions were extracted. Two authors (ES and TN) extracted relevant data from each paper. Two other authors (PD and WA) performed quality checks to ensure accuracy and completeness. Discrepancies during the extraction process were discussed with these 4 authors. The quality appraisal and risk of bias for the included studies were assessed using two distinct tools: (1) the second version of the Cochrane Risk of Bias tool for randomized trials and (2) the Risk of Bias In Non-Randomized Studies of Interventions-I tool for quasi-experimental studies. Each domain was categorized as low, moderate, serious, critical, or no information [31]. Both tools used the scores from these domains to determine the overall risk of bias [31,32].

## Results

### Overview

**Figure 1:**
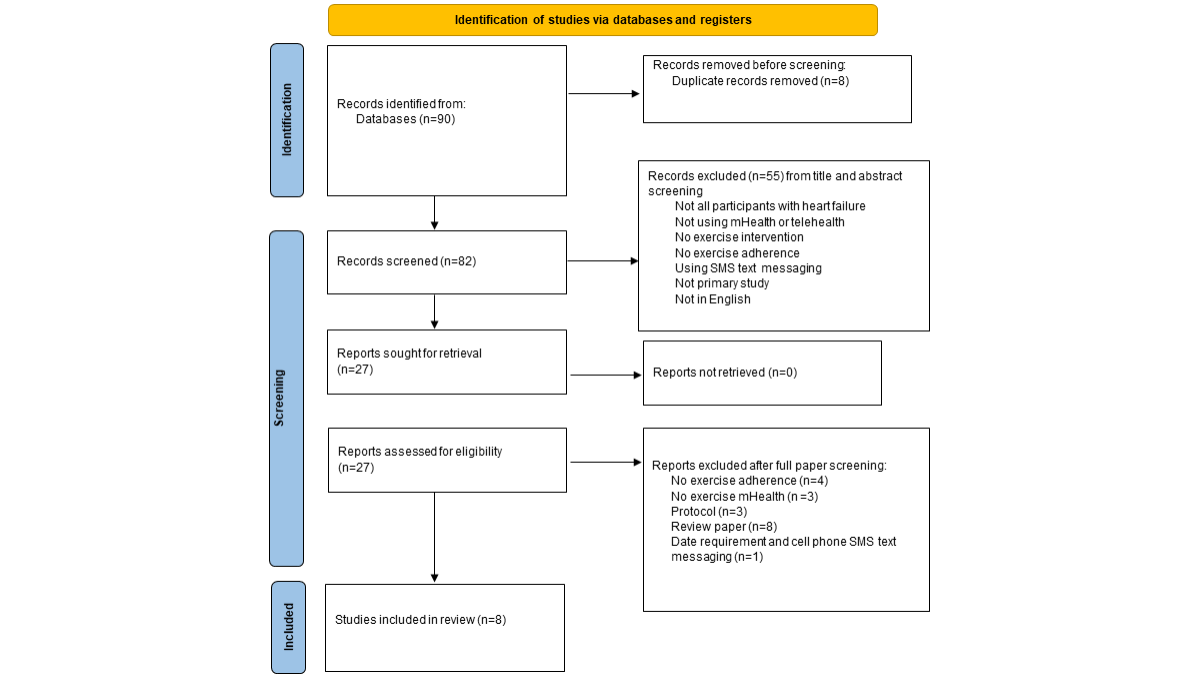
PRISMA diagram detailing the screening process.

Our initial search yielded a total of 90 papers. The removal of duplicates yielded 82 papers to screen. In total, 55 papers were extracted during the first screening process, resulting in 27 papers being sought for full-text retrieval. The 55 papers did not meet inclusion and exclusion criteria from the title and abstract screening for not including participants with HF, not using mHealth or telehealth intervention, not using an exercise intervention, not reporting adherence, using cell phone SMS text messaging for intervention, not a primary study, and not published in English. Following the second screening process, 19 papers were excluded for the following reasons: review papers (n=8), did not report adherence (n=4), no exercise intervention using mHealth (n=3), papers reporting protocols (n=3), and papers did not meet date requirements (n=1). Only 8 papers were included for data extraction. The PRISMA diagram (Figure 1) highlights the screening process. The papers included 4 randomized controlled trials (RCTs) [33-36] and 4 quasi-experimental trials [37-40]. A total of 4 studies are from Asia [33,34,37,38], 3 from the United States [35,39,40], and 1 from Australia [36]. Descriptions of these studies are provided in Table 1.

**Table 1:** Study location, study design, sample characteristics including participant’s New York Heart Association (NYHA) classification, ejection fraction, age, recruitment setting, and reports of baseline evaluation of exercise adherence.

Author (year), country	Design (groups, n); length of study	Sample size, n; sex; race	NYHA classification; ejection fraction, mean (SD); age (years), mean (SD)	Recruitment site	Baseline evaluation of exercise adherence
Tsai et al (2022) [37], Taiwan	Quasi-experimental (2); 6 months	81 (intervention=40; control=41)	Not reported; 33.5% (11.12%); 73.3 (5.0)	Hospital prior to discharge	Not reported
Nagatomi et al (2022) [33], Japan	RCT^a^ (2); 3 months	30 (intervention=15; control=15); male=53%; Asian	Class II-III; 42.2% (17.4%); 63.7 (10.1)	Outpatient clinic	Engaged in outpatient rehabilitation >2 times per week
Liu and Liu (2022) [34], China	RCT (2); 12 weeks	60 (intervention=30; control=30); male=17, female=13; Asian	Class II-III; 36.02% (5.12%); 53.27 (7.1)	Hospital prior to discharge	Not reported
Kikuchi et al (2021) [38], Japan	Quasi-experimental (1); 12 weeks	10; male=55%, female=45%; Asian	Class II-III; 40% (not reported); 76 (7)	Hospital prior to discharge and outpatient	Good (more than 90 minutes a week), fair (30-90 minutes a week), and poor (less than 30 minutes a week)
Deka et al (2019) [35], United States	RCT (2); 8 weeks	30 (intervention=15; control=15); male=63%; female=37%; White=100%	Class I-III; 41% (12.6%); 61.7 (11.6)	Outpatient clinic	Self-reported; excluded if >2 days per week of 30 minutes exercise
Lloyd et al (2019) [39], United States	Quasi-experimental (1); 1 month	12; not reported	Class II-III; 40% (not reported); 67 (not provided)	Prior to hospital discharge	Not reported
Hwang et al (2017) [36], Australia	RCT (2); 8 weeks	53 (intervention=24; control=29); male=75%; female=25%	Class I-III; 35% (17%); 67 (12)	Prior to hospital discharge	Not reported
Donesky et al (2017) [40], United States	Quasi-experimental (2 nonrandomized groups); (12 weeks)	15 (intervention=7; control=8); male=43%; female=57%	Class II-III; not reported; 71 (8.5)	Outpatient clinic	Not reported

^a^RCT: randomized controlled trial.

### Study Characteristics of mHealth Interventions for Exercise Adherence in HF

This review builds on research to describe study characteristics of mHealth interventions for exercise adherence in HF, including details of sample demographics, sample sizes, exercise programs, and theoretical frameworks. As detailed in Table 1, the sample sizes ranged from 12 to 81, with study durations lasting 4 to 26 weeks. The studies from Asia included Asians in their study, and the studies from the United States and Australia primarily included White patients with HF. While 2 studies had a larger proportion of male participants (63% and 75%) [35,36], other studies seem to have an equal representation of male and female participants. One study did not report the race of the participants enrolled in the study [39]. The mean ages of the participants included in the studies range from 53 to 73 years. The NYHA classifications ranged from class I to class III, with mean ejection fractions ranging from 33.5% to 45%. One study did not report the ejection fraction of the participants [40]. In total, 3 studies recruited participants from a cardiac outpatient clinic [33,35,40], while 5 studies recruited participants from an inpatient hospital setting [34,36-39].

With exercise being performed at home, baseline training for patients with HF to safely perform exercise is important. One study incorporated 1 week of center-based cardiac rehabilitation exercise training before transitioning to a home setting [37]. Two studies provided 1 training session on the exercise program at baseline [35,38]. One study indicated that the participants received family cardiac rehabilitation prior to being recruited [34]. Among the studies that were RCTs, 1 study reported that the control group was provided the same exercise prescription as the intervention group [35], and 1 study provided the control group with family cardiac rehabilitation training guided by mobile medical technology [34]. Other studies did not provide details of the number of sessions needed to train the participants in the prescribed exercise program.

For exercise modality, 1 study used a yoga program [40], 1 study used a walking program [35], 1 study combined jogging with walking [34], 1 study used a cycle ergometer [38], 2 studies combined walking with cycle ergometry [33,37], and 1 study used a stepper [39]. One study did not provide details of the aerobic component of the exercise program [36]. Two studies added a resistance exercise component to the exercise prescription [33,36]. One study used multiple types of aerobic exercises (such as walking or using a cycle ergometer, a stepper, and an elliptical) and incorporated 1 day per week of facility-based exercise [37]. Studies that prescribed a primarily home-based exercise prescription used a walking or cycle ergometer program.

Rating of perceived exertion (RPE) was the most used tool for regulating the intensity of exercise [33-37]. Overall, participants in these studies were asked to keep their RPE<14 on the Borg (6-20) RPE scale, which is suggestive of moderate-intensity exertion [41]. Additionally, a few studies also used heart rate for moderate exercise intensity regulation. These strategies to regulate intensity using heart rate included keeping the exercise heart rate at resting heart rate+30 beats per minute [37], resting heart rate+(maximum exercise heart rate–resting heart rate)×0.4 [34], and below maximal heart rate during baseline training on the exercise program [35]. Three studies did not provide details on how intensity of exercise was regulated [38-40].

Prescribed exercise durations were between 30 and 60 minutes. The exercise prescriptions used by the studies varied but ranged from 3 to 5 days per week. While 1 study advised participants to exercise daily [39], 2 studies recommended 2 days per week of exercise [36,40]. One study did not report on the frequency of recommended exercise per week [37]. We found that the exercise prescriptions in most studies were investigator-developed programs. Reference to the recommended guidelines of 150 minutes per week of moderate-intensity aerobic exercise for patients with HF was done in 1 study [35].

Only 1 study indicated developing their intervention using a theoretical platform [35]. The study used concepts from Bandura’s self-efficacy theory and Ajzen’s theory of planned behavior to bring about change in adherence behavior. The other studies did not specifically mention any theory.

### Summarize Types of mHealth Technology Used to Improve Exercise Adherence in Patients With HF

Table 2 provides information about the mHealth technologies used and how they were incorporated into the intervention. As summarized in Table 2, videoconferencing was used in 4 studies [35,36,38,40], while 4 studies [33,34,37,39] used mobile apps for the devices used in the study. The apps were installed in smartphones (n=3) [33,34,37], laptops, tablets, or desktop computers using Wi-Fi or 3G wireless broadband connection (n=4) [35,36,38,39], and 1 study used a television that was connected to the internet [40]. Studies published in the past 3 years used smartphone-based apps, while studies that were a little older used more laptops, computers, and tablets. In the 4 studies that used a videoconferencing platform, videoconferencing was delivered using commercially available software such as Vidyo (Vidyo Inc) [35], Adobe Connect 9.2 [36], DocBox (DocBox, Inc) [40], and the Remohab integrated rehabilitation platform (Remohab) [38]. Two studies used a Fitbit activity monitor along with the Fitbit app [33,35]. Two studies used apps to transfer information to the physician’s office via the cloud database [33,37]. Three studies using videoconferencing included interventions that were in a group setting [35,39,40]. One study used an app that provided voice prompts during exercise [34]. While 1 study simply engaged participants in cohorts (n=5) for a discussion session for social support [35], 2 studies focused on delivering an exercise program that included yoga (n=8) [40] and aerobic exercise (n=4) [36].

**Table 2:** mHealth technology used and details of the study intervention^a^.

Study author(s) (year)	Type of mHealth technology used	Type of software	Devices used	Details of exercise intervention (individual or group)
Tsai et al (2022) [37]	App	HF^b^ health management mobile app system platform	Smartphone	The exercise parameters were recorded on the HF health management mobile app system platform by each patient and daily transmission to the hospital’s cloud database as home-based cardiac telerehabilitation. Monitoring parameters include body weight, blood pressure, resting heart rate, exercise heart rate, exercise time, and abnormal symptom signs.
Nagatomi et al (2022) [33]	App	Fitbit	Fitbit Inspire HR worn on nondominant hand; smartphone	Comprehensive home-based cardiac rehabilitation program that combines patient education, exercise guidance, and nutritional guidance using information and communication technology. Messages sent once a week through the Fitbit app or by telephone. Video instructions on performing exercises were provided using a QR code via smartphone.
Liu and Liu (2022) [34]	App	Exercise rehabilitation app software designed by the family cardiac exercise rehabilitation training research team	Android smartphone	The app provided exercise guidance via voice prompts on current exercise speed, heart rate, acceleration or deceleration reminder, exercise rhythm adjustment, etc.
Kikuchi et al (2021) [38]	Videoconferencing and remote monitoring	Integrated rehabilitation platform (Remohab Inc)	An Internet of Things–equipped ergometer (Charimo, Remohab Inc), an Android-compatible tablet (TAB3-X70L, Lenovo), and a wireless electrocardiographic monitoring device (hitoe, Toray)	In total, 30 minutes to install the system including Wi-Fi at the participants home and 60 minutes to instruct the use of the platform. Exercises were done individually. Participants exercised on a cycle ergometer for 30 minutes or less while videoconferencing with a nurse. For each session, exercise intensity was set by the attending physician. There was live monitoring of heart rate and electrocardiogram for arrhythmia that was transferred via Wi-Fi to the physician’s office.
Deka et al (2019) [35]	Videoconferencing and app	Vidyo and Fitbit app	Fitbit Charge HR; tablet, computer, laptop; home Wi-Fi or wireless connectivity	Education on HF self-care on the following topics: understanding HF, exercise and activity with HF, how to follow a low sodium diet, HF medication, dealing with HF symptoms, depression and anxiety with HF, and managing lifestyle changes. Discussion on the previous week’s adherence, barriers, and facilitators. Five participants in each cohort.
Lloyd et al (2019) [39]	App	REDCap^c^	Tablet computer with wireless connectivity	A 5-minute video with an older adult performing the intervention tasks was created and provided to participants. Feedback was provided in the form of personal data graphs.
Hwang et al (2017) [36]	Videoconferencing	Adobe Connect 9.2	Laptop computer, mobile broadband device connected to 3G wireless broadband internet	Physical therapist–guided telerehabilitation exercise in groups (up to 4 participants). In total, 15 minutes were spent at the start of each session on a discussion on educational topics that replicated a center-based program.
Donesky et al (2017) [40]	Videoconferencing	DocBox	The DocBox was connected to the participants’ home television for live-streaming yoga classes	Group teleyoga. Postures included mountain, half-down dog, cat, triangle, supported bridge, simple twist, staff, corpse, and cobbler poses, with postures modified as needed to meet the physical ability of each participant. Yoga classes were designed to integrate breathing exercises (slow breathing and extended exhalation breathing), imagery, meditation, and relaxation.

^a^ mHealth: mobile health.

^b^HF: heart failure.

^c^REDCap: Research Electronic Data Capture.

### Adherence: Description, Measurement and Achievement

In our review, we found that, in the majority of studies, the term adherence was used to measure “adherence to a study-specific exercise program.” Only 1 study used the term adherence in reference to the recommended exercise guidelines [35]. As seen in Table 2, all studies relied on an objective tool for measuring the amount of exercise to calculate adherence. In total, 2 studies used a Fitbit [33,35], 3 studies used a telemonitoring platform [34,37,38], and 2 studies directly observed exercise being performed during the videoconferencing session [36,40].

Table 3 highlights the details of the exercise program and exercise adherence achieved in the studies included in the review. Comparing exercise adherence among the different studies is difficult because of the variance in the exercise program. While most studies reported adherence in proportions of participants achieving adherence, 2 studies reported adherence in mean minutes of exercise achieved by the intervention group [35,39].

**Table 3:** Details of the exercise program and exercise adherence.

Study (author, year)	Exercise setting; individual or group	Modality	Intensity	Duration	Frequency	Length; attrition	Adherence calculation	Adherence achieved	Adherence sustainability report
Tsai et al (2022) [37]	1 time per week outpatient cardiac rehabilitation and home-based exercise telemonitoring	Cycle ergometers, stepper, elliptical, walking	RHR^a^+30 bpm or <13/20 RPE^b^	40-60 minutes	Not reported	6 months; n=4	Not reported	Intervention group: 95.2% adherence	None
Nagatomi et al (2022) [33]	Home only	Stretching and resistance training using weights and walking or cycle ergometry	11-13 RPE	Aerobic: 30-40 minutes	Aerobic=3-5 times per week; resistance=2-3 times per week	3 months	Percentage of participants who exercised 4 days per week; exercise performance rate=number of exercise days/total number of intervention days	Adherence was 73%; 11 participants achieved >4 days of exercise	None
Liu and Liu (2022) [34]	Home only	Walking, jogging	THR^c^=RHR+(exercise HRmax^d^–RHR)×0.4; Borg scale also used, target RPE not reported	30-60 minutes	3-5 times per week	12 weeks	80% for good adherence, 50%-79% for compliance, and below 50%	Intervention group: adherent 12, partial adherent 10, and poor adherent 8; control group: adherent 5, partial adherent 12; and poor adherent 13	None
Kikuchi et al (2021) [38]	Home only	Cycle ergometry	THR calculated from CPET^e^; specifics not provided	30 minutes or lower	3 times per week	12 weeks; n=1	Rate of attendance of a total of 36 offered sessions	94.4%	None
Deka et al (2019) [35]	Home only	Walking	RPE 11-14; heart rate during baseline walking	30 minutes	5 times per week	8 weeks; none	Self-reported exercise validated using Fitbit; ([actual number of minutes per week]/[150 minutes per week target goal]×100), adherent: >80%, partially adherent: 20%-80%, and nonadherent: <20%	Intervention group: 88 minutes per week; control group: 86 minutes per week	None
Lloyd et al (2019) [39]	Home only	Aerobic stepper	Not reported	Not reported	Daily	1 month	Self-reported number of minutes of exercise per day	Use of stepper was consistent; overall mean minutes of exercise increased by 2.4 minutes; exercise minutes ranged from 0 to 21 minutes	None
Hwang et al (2017) [36]	Center-based for the control group and home-based for the intervention group; group exercise: 4 in each group	Aerobic and resistance	RPE 9-13	40 minutes of aerobic and resistance	2 times per week	12 weeks	Adherent: >80% of sessions attended, partly adherent: 20%-80% of sessions attended, and nonadherent: <20%	Intervention group: 49 adherent or partially adherent	After 12 weeks
Donesky et al (2017) [40]	Home only	Yoga	Not reported (mean heart rate stayed below 90 bpm)	35 minutes of poses	2 times per week	8 weeks	Attendance to number of sessions	90% attendance to classes	None

^a^RHR: resting heart rate.

^b^RPE: rating of perceived exertion.

^c^THR: target heart rate.

^d^HRmax: maximal heart rate.

^e^CPET: cardiopulmonary exercise testing.

In 2 studies, adherence was the primary outcome [35,40]. While one study investigated adherence to a walking program [35], the other study reported adherence to a teleyoga program [40]. The primary outcomes in the other studies included exercise intolerance [42], medication adherence [39], exercise tolerance [34,37], and physical function [33].

Similar to our previous review [26], we found that even in studies using mHealth technology, exercise adherence was not a primary outcome and mostly reported adherence as a secondary outcome measure. As previously noted, measurement of adherence requires a study design that extends beyond the intervention phase to determine the effectiveness of the intervention in bringing about lifestyle changes and sustainability of adherence behavior. We found this to be lacking in all studies in this review. Most studies included in this review were exploratory in nature and were testing for feasibility, acceptability, or initial pilot testing of the intervention design or exercise protocol.

### Effect of demographic and clinical characteristics on Exercise Adherence

No study reported the effect of age, sex, race, HF etiology, NYHA functional classification, and HF etiology on exercise adherence. We found most studies included a sample of patients with HF who had homogenous demographic and clinical characteristics. In addition, the small sample sizes and quasi-experimental design prevent from making any conclusion on adherence based on age, sex, race, NYHA classification, and HF etiology.

### Risk of Bias

Tables 4 and 5 detail the risk of bias assessments for the included studies. For the 4 RCTs evaluated using the Risk of Bias 2 tool, 1 was classified as having a low risk of bias, 1 as having a moderate risk, and 2 as having a high risk. The moderate and high risks of bias were attributed to bias from the randomization process (n=4, 50%), deviations from intended interventions (n=2, 25%), missing outcome data (n=2, 25%), and outcome measurement (n=2, 25%). Among the 4 quasi-experimental studies assessed using the Risk of Bias In Non-Randomized Studies of Interventions-I tool, 2 were rated as having a moderate risk of bias, and 2 had a serious risk of bias. The moderate and serious risks stemmed from confounding variables (n=2, 25%), classifications of interventions (n=2, 25%), deviations from intended interventions (n=2, 25%), missing data (n=6, 75%), and outcome measurement (n=4, 50%).

**Table 4:** Assessment of study risk of quasi-experimental papers.

Author(s) (year)	Confounding	Selection	Classifications	Deviations from interventions	Missing Data	Measurement	Selection of reported result	Overall bias
Donesky et al (2017) [40]	Low risk	Low risk	Low risk	Low risk	Moderate risk	Moderate risk	Low risk	Moderate risk
Lloyd et al (2019) [39]	Low risk	Low risk	Low risk	Low risk	Moderate risk	Serious risk	Low risk	Serious risk
Kikuchi et al (2021) [38]	Low risk	Low risk	Low risk	Serious concern	Moderate risk	Low risk	Low risk	Serious risk
Tsai et al (2022) [37]	Moderate risk	Low risk	Moderate risk	Low risk	Low risk	Low risk	Low risk	Moderate risk

**Table 5:** Assessment of study risk of randomized controlled trial papers.

Author(s) (year)	Arising from randomization process	Deviations from intended interventions	Missing outcome data	Measurement of the outcome	Selection of the reported results	Overall bias
Hwang et al (2017) [36]	High risk	Moderate concerns	Moderate risk	Low risk	Low risk	High risk
Deka et al (2019) [35]	Low risk	Low risk	Low risk	Moderate risk	Low risk	Moderate risk
Liu and Liu (2022) [34]	Low	Low risk	Low risk	Low risk	Low risk	Low risk
Nagatomi et al (2022) [33]	High risk	Low	Low risk	Low risk	Low risk	High risk

## Discussion

### Principal Findings

Decades of research have established the benefits of regular exercise in patients with HF [43]. However, adherence to recommended exercise for patients with HF is often hampered by their clinical condition and other structural and sociodemographic barriers [44]. With significant improvements in wireless internet connectivity and smartphone use across the world, mHealth-driven interventions hold immense promise with the ability to deliver the intervention to the participant’s home using varied forms of multimedia engagement tools [45,46]. This review found 8 feasibility or pilot studies delivering exercise interventions using mHealth tools and reporting on adherence to varied exercise programs in patients with HF. Studies had a moderate to high risk of bias, and theoretically sound and fully powered RCTs were found to be lacking, making it difficult to determine clinical efficacy and the effect of demographic and clinical characteristics on adherence.

Previous studies have highlighted the variation in how the concept of adherence has been used by researchers reporting adherence to exercise in patients with HF [26]. Similar to prior studies, our review found the term “adherence” used primarily to report “attendance” to the investigator-developed program [23]. While this approach is good for assessing the effectiveness of the study intervention, researchers should clarify how adherence achieved in their study translates into meeting adherence to the recommended exercise guidelines for patients with HF. A position statement from the Heart Failure Association from the European Society of Cardiology has categorized exercise adherence as fully adherent=achieving >80% of exercise recommendations, partially adherent=achieving 20%-80% of exercise recommendations, and nonadherent=achieving <20% of exercise recommendations [44,47].

The earlier-mentioned adherence categorizations have also been borrowed from the medication literature [47]. It needs to be acknowledged that the complexity and challenges associated with adherence to exercise are different compared to adherence to other types of self-care (eg, medication adherence). To achieve exercise adherence, the extent of lifestyle and behavioral changes needed to be made by a patient with HF, experiencing frequent peaks and troughs in symptoms, can be challenging and also different from patients with other chronic clinical conditions such as diabetes and hypertension [25]. A systematic review and meta-analysis showed substantial heterogeneity in exercise adherence rates and dropouts across clinical populations [25]. This heterogeneity, along with the varied ways adherence is measured [48], makes it even more challenging to compare adherence rates within and across populations. Self-efficacy, motivation, enjoyment, and attention to exercise relapse management are important factors associated with physical activity in patients with HF [49-51]. Interventions should target not only building exercise self-efficacy but also ensuring that participants are enjoying the mHealth experience and the exercise program for maintaining motivation and achieving sustained adherence over time. Adults with HF often experience exacerbation of their symptoms that impede their ability to exercise. Relapse management strategies to reinitiate exercise after a break are an important component of interventions to promote exercise adherence.

As expected, all studies delivered their mHealth interventions to the participants’ homes. The more recent studies adopted more smartphone-based interventions. This observation makes sense as, in the past few years, internet and smartphone access and use have increased significantly globally [52] and among patients with HF [53]. Most exercise prescriptions used in the studies in this review were investigator-developed programs. Alternate forms of exercise (such as yoga) have been delivered using mHealth technology; however, guidelines for such forms of alternate exercise for patients with HF have not yet been standardized, and their benefits have also not been clearly established. Due to the varied exercise programs prescribed in the different studies, it is difficult to compare adherence achieved between the studies. As such, it is also difficult to compare study results, as exercise and health outcomes have a dose-dependent relationship [54,55]. mHealth intervention can be challenging, as it requires training participants to use the mHealth technology in addition to the exercise program. The complexity of the technology can make it difficult for patients with HF, who tend to be older adults, to not be able to use the technology as directed, thereby presenting issues with internal validity for the research study and also potential dropout. Six studies mention providing 1 baseline training session with additional technical help if needed over the phone or in person [34-36,38-40]. No study provided details of challenges encountered in training participants to use the technologies that were used. The studies included in this review did not report any safety concerns or adverse incidents associated with the exercise performed in a home setting. Exercise training at baseline, depending on the simplicity of the exercise program, ranged from 1 training session to prior enrollment in a cardiac rehabilitation program. As studies using mHealth technology are likely to be delivered to the participants’ homes and mostly unsupervised, assessment of the ability of patients with HF to safely exercise on their own is important. While a majority of studies reported achieving good exercise adherence, the intervention period was short (lasting 1-6 months), and the sustainability of exercise behavior was not measured. We also noted a lack of and a need to determine the utility and effectiveness of theoretically sound mHealth-driven interventions across different strata of patients with HF, including age, sex, and race.

In our previous review, we highlighted that exercise diaries were the most commonly used tools for measuring exercise adherence in HF studies reporting adherence [26]. One advantage of using mHealth technology is also the ability to collect objective exercise data rather than relying on self-reported data, which can be erroneous due to overestimation [56]. The use of physical activity monitoring devices such as Fitbits allows for measuring and remotely monitoring objective physiological parameters, such as heart rate and step count, during exercise. Most studies included in this review prescribed simple exercise programs that included walking, cycle ergometer, or stepper, with a couple of studies adding resistance training to their exercise program. The use of RPE (eg, Borg scale) was the most used tool for regulating exercise intensity. Subjective tools are easy to use and are often preferred by clinicians and patients. However, the heart rate function in commercially available physical activity monitors such as Fitbit offers more precise exercise intensity regulation and was used by some studies. Research shows that patients with HF like using the heart rate and step count feature in Fitbit [35]. It is important that patients with HF, who tend to be older adults, are adequately trained in using these devices to prevent potential dropouts from facing technological challenges.

### Limitations

This review included studies that were published between 2015 and 2022. We completed a thorough search, but some papers may have been missed and not included in the review. The study is limited to the use of mHealth for exercise adherence in patients with HF and does not include the use of mHealth technologies for improving other self-care recommendations. There is a risk of bias on the part of the authors, which we have tried to mitigate through our search strategies, inclusion, exclusion, and screening process outlined earlier.

### Future Recommendations

Our review shows preliminary evidence of using mHealth technology for targeting exercise adherence in patients with HF. However, there is a lack of theoretically sound and fully powered RCTs to suggest its effectiveness. It is crucial for studies using mHealth technology to improve study quality and limit the risk of bias, as noted in this review, particularly by limiting confounding factors. In addition, it is important to determine the sustainability of exercise behavior after the intervention period when the intervention stimuli have been removed. How adherence is measured and reported also needs consideration. Because adherence has mostly been reported to investigator-developed programs, it is difficult to compare the adherence achieved in different studies. As such, we suggest that adherence achieved to investigator-developed exercise programs should be standardized to the recommendations for exercise for patients with HF to help compare the effectiveness of different programs. Reporting the proportion of participants achieving adherence may be better than reporting the mean minutes of exercise performed by participants, as the mean calculations are influenced by outliers. Tracking daily exercise using mHealth tools and reporting changes in the number of minutes of exercise from baseline can provide a better picture of the effectiveness of home-based interventions, especially in patients with HF who experience peaks and troughs in symptoms. They can also be useful in validating self-reported exercise and physical activity [57]. Considering that making lifestyle changes is challenging for many patients with HF, the practical and clinical significance of the adherence achieved using mHealth interventions should also be highlighted alongside reports of statistical significance. Finally, the feasibility and acceptability of mHealth technology across different races, sexes, and cultures needs to be studied. mHealth provides opportunities to explore culturally tailored interventions in HF that have been grossly underinvestigated.

### Conclusions

There is some preliminary evidence suggesting the feasibility of using mHealth technology for building exercise adherence in patients with HF; however, theoretically sound and fully powered studies, including studies on minoritized communities, are lacking. In addition, lacking is a report on the sustainability of the achieved adherence beyond the intervention period. The study provides some areas for researchers to focus on in future studies.

## Appendix

Multimedia Appendix 1 PRISMA-S Checklist for Reporting Literature Searches in Systematic Reviews.

## Abbreviations

HF: heart failure
mHealth: mobile health
NYHA: New York Heart Association
PRISMA: Preferred Reporting Items for Systematic Review and Meta-Analyses
RCT: randomized controlled trial
RPE: rating of perceived exertion

## Data Availability

Data sharing is not applicable to this paper as no datasets were generated or analyzed during this study.
